# Calmodulin: The Essential Biological Regulator Self-Regulates Its Activity, Structure and Function

**DOI:** 10.3390/biology15191750

**Published:** 2026-10-02

**Authors:** Danton H. O’Day

**Affiliations:** 1 Department of Biology, University of Toronto Mississauga, Mississauga, ON L5L 1C6, Canada; danton.oday@utoronto.ca; 2 Cell and Systems Biology, University of Toronto, Toronto, ON M5S 3G5, Canada

**Keywords:** calcium regulation, calmodulin-binding domains, CaMKII, calcineurin, calmodulin hypothesis, *CALM* genes, CAMTAs, post-translational modifications, self-regulation

## Abstract

Calmodulin is an evolutionarily conserved critical controller protein found in animals, plants, fungi, and microbes. As the primary calcium sensor and effector in eukaryotes, calmodulin translates calcium signals into vital biological responses by binding to and regulating calmodulin-binding proteins. While it was previously shown to control several hundred proteins, evidence now indicates it binds to over 100 in its calcium-free state and more than 500 when activated by calcium ions. While calmodulin function is modulated by calcium levels, the final cytosolic levels of calcium are often under calmodulin’s control. Human calmodulin is encoded by three unique genes (*CALM1–3*), each of which produces an identical functional protein. The *CALM* genes are among the most mutation-intolerant sequences in the human genome. They are under the control of two calmodulin-binding transcription activators (CAMTAs) that are also ancient, highly conserved, and, as their name implies, under the control of calmodulin. Thus, calmodulin controls the transcription of the genes that encode it. Finally, two primary post-translational events that affect the function and stability of calmodulin are also regulated by it: methylation, which stabilizes the functional protein, and phosphorylation, which regulates its activity as needed. This review covers those topics to reveal that calmodulin controls its own expression, structure, and function, reflecting its unique position as an essential major regulator of cell function.

## Simple Summary

Present in all eukaryotes from microbes to humans, calmodulin is a vital protein that binds to and regulates more targets than any other protein. An essential calcium-binding protein, calmodulin is highly conserved and functionally identical across species. This review begins with a basic overview of its structure, function, and unique regulatory mechanisms. It then presents, for the first time, arguments revealing that this overseer of core cellular processes not only controls its own levels but also its ability to bind to and regulate its different targets. The genes encoding calmodulin self-regulate its cellular levels. The resultant protein then controls post-translational modifications to itself that affect its structure and regulatory abilities. To precisely control cellular events, calmodulin binds to and regulates multiple calcium ion channels and receptors, ensuring the levels of divalent cations meet its functional needs. This review emphasizes that this combination of unique attributes of calmodulin further supports its unique and essential role in cell structure and function. Considering this and its key roles in multiple diseases, including cancer, Alzheimer’s, and other neurodegeneration, more research is warranted.

## 1. Introduction

For over 40 years, calmodulin (CaM) has been recognized as a universal calcium sensor and regulator of cell function [1]. Subsequent research has continued to support this idea. The innumerable biological events controlled by CaM include, but are not limited to, smooth, cardiac, and skeletal muscle contraction; cytoskeletal dynamics in cell movement; the intracellular movement of proteins and organelles along microtubules; neurotransmitter release, the inflammatory response, and metabolism [2,3,4]. Because of this, CaM is also a central regulator in various diseases, including a wide variety of cancers and neurodegenerative diseases (NDDs), among others [5,6]. Because of its essential nature, maintaining CaM’s levels and functionality is critical. Here we take a unique approach to understanding CaM by examining how this major regulator controls its own availability, structure, and functionality to ensure essential cell functions are maintained.

## 2. Calmodulin General Functions

CaM is routinely referred to as a calcium sensor and effector, but it is more than that. The literature is full of original research articles and reviews that cite vital functions for CaM, yet, to date, no attempt appears to have been made to list them all. Here we will list a few basic events and the CaM-binding proteins (CaMBPs) that are involved. The extensively reviewed calcium–calmodulin-dependent protein kinase II (CaMKII) and calcineurin stand out as major event regulators [7,8]. Working together or independently, these enzymes serve as a primary control system for innumerable events including neuronal (learning, memory, plasticity, neurotransmission) and non-neuronal processes (heart rate, insulin secretion, glucose uptake, cell cycle progression). Three other examples include MLCK (myosin light-chain kinase), NOS (nitric oxide synthase), and neurogranin. MLCK phosphorylates myosin, an essential event in smooth muscle contraction, cell motility, and other events. Smooth muscle contraction mediates peristalsis that pushes food and waste through our digestive system. In the cardiovascular system, it regulates blood pressure by controlling blood vessels’ diameter; in the respiratory system, it similarly controls bronchioles, and in the urinary and reproductive systems, it mediates voiding of the bladder, sperm motility, and oversees uterine contractions during childbirth [9]. The three forms of NOS generate nitric oxide that is involved in blood vessel relaxation and penile erection [10]. In the brain, it acts as a neurotransmitter involved in synaptic plasticity, learning, and memory [11]. As part of the immune response, it triggers cytokines, fights off infections, and kills tumor cells. In the blood, it prevents platelets from sticking together. Neurogranin has neuronal, cardiovascular, and endothelial functions [12,13]. It tethers CaM, making it available for neuronal functions to control long-term potentiation, strengthening neuronal connections and encoding new information [14]. It serves as a biomarker for neuronal health in neurodegenerative diseases and, in the heart, it is involved in cardiomyocyte control of calcium levels, while in endothelial cells it oversees mitochondrial health and redox levels [15,16,17].

In 2000, the comprehensive Calmodulin Target Database listed over 300 CaM-binding sequences (http://calcium.uhnres.utoronto.ca/ctdb/; 7 August 2026) along with a search program for determining if any of the known Ca^2+^-CaM-binding motifs are present in a protein sequence [18]. It had long been accepted that CaM binds to 300+ CaMBPs, but current research suggests that over 600 proteins are targets: 100+ apo-CaM and 500+ Ca^2+^-CaM-binding proteins [19,20,21]. With over 600 binding proteins, the previous information does not come close to revealing just how pivotal CaM is to the functioning of cells, tissues, and organs in the human body. A short overview of some specific functions mediated by CaM will add more insight. While its role as a calcium sensor and buffer is well known, its function in binding to and regulating the primary toxic biomarkers of Alzheimer’s, Parkinson’s, and Huntington’s diseases (i.e., Aβ, Tau, αSyn, and Htt) has only come to light more recently [15,22,23,24,25,26,27]. One of the most well-studied examples of enzyme activation by CaM is CaMKII, but activation of SK ion channels is also well documented [28,29]. CaM can also be inhibitory, as revealed by its effects on enzymes (e.g., phosphoglycerate kinase, PGK), receptors (e.g., G protein-coupled receptor kinases, GRKs) and ion channels (e.g., TRPV5/6) [30,31,32]. Depending on calcium levels and other factors, CaM acts as a switch to activate and deactivate receptors (e.g., inositol 1,4,5-trisphosphate receptor, IP3R) and transcription factors (e.g., calmodulin-binding transcription activators, CAMTAs; E-protein subclass of bHLH transcription factors, SEF2) [3,33,34]. It mediates protein dimerization (e.g., estrogen receptors), protein translocation (e.g., Simian Virus 40 (SV40) large T-antigen), and protein scaffolding (e.g., nuclear receptor interaction protein, NRIP; Ras GTPase-activating-like protein, IQGAP) [35,36]. CaM can also control its own localization (e.g., Ng) and posttranslational modification via phosphorylation (casein kinase II) and methylation (e.g., methyltransferase) [14,36,37]. Aspects related to CaM’s control of its own levels, structure, and function are covered in more detail below.

## 3. Calmodulin Structure and Target Binding

**Figure 1:**
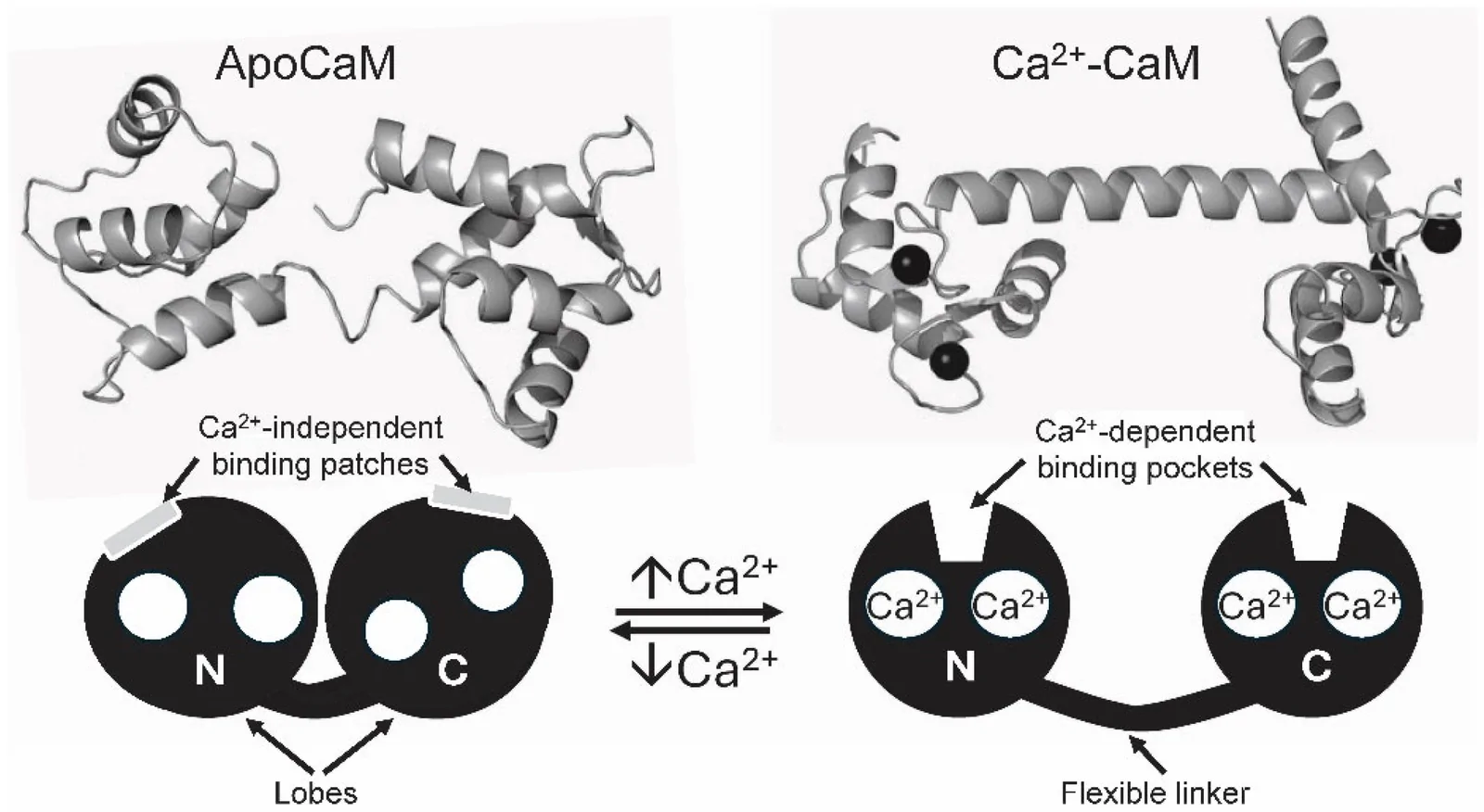
Basic structure of apoCaM versus Ca^2+^-CaM revealing the binding regions for calcium-free and calcium-dependent target binding. The **upper** ApoCaM and Ca^2+^-CaM images depict single aspects of their 3D structures. The **lower** illustrations demonstrate the presence of calcium-independent binding patches and calcium-dependent binding pockets as detailed in the main body of the review.

CaM (148aa, 16.7 kDa) is a small calcium sensor and regulatory protein consisting of two terminal calcium-binding lobes joined by a flexible alpha helix as diagrammed in Figure 1 [28,38]. The N- and C-lobes each possess two EF-hand type Ca^2+^-binding sites such that up to four calcium ions can bind, two in each lobe. In its condensed calcium-free state, apoCaM possesses some structural attributes that allow the flexible linker to assist in rapid target searching and binding via hydrophobic patches on each lobe [39,40]. Calcium-independent binding by apo-CaM relies on pre-formed, partially open structural surfaces in these patches that mediate electrostatic and shallow hydrophobic interactions, especially with target IQ motifs [39].

On the other hand, canonical calcium-dependent binding is mediated by methionine-rich pockets that open in the presence of calcium ions [41]. Depending on the calcium concentration, Ca^2+^-CaM undergoes a marked change in shape, converting the condensed form to a barbel-shaped form while simultaneously opening the calcium-dependent binding pockets in each lobe that mediate binding to specific motifs within target protein CaM-binding domains (CaMBDs) (Figure 1). As detailed below, CaMBD motifs comprise bulky, hydrophobic anchoring residues that fit into the hydrophobic pockets of the two CaM lobes [41]. The pockets have a defined sequence of four large hydrophobic amino acids: phenylalanine, leucine, and methionine, or FLMM. The N-terminal hydrophobic FLMM pocket consists of Phe19, Leu32, Met51, and Met71, while the C-terminal hydrophobic pocket consists of Phe92, Leu105, Met124, and Met144 [41,42].

The opening of the hydrophobic pockets in each lobe is affected by calcium levels, with the C-lobe binding to the cation with an approximately 10-fold higher affinity than the N-term lobe (N-lobe), differences that affect the sequence and effect of target binding [43].

CaM binds to and regulates its targets in a variety of ways. ApoCaM binding is primarily mediated via classic IQ motifs (basic, IQxxxRxxx; full, IQxxxRKxxxxRKxxx) and variants (e.g., ([IVL]QxxxRxxxx[RK]xx[FILVWY])) [20,28]. In contrast, Ca^2+^-CaM binding can occur in many ways. Classic or canonical Ca^2+^-CaM binding involves motifs of anchoring hydrophobic amino acids surrounded by a net positive charge (basic), which is electrostatically attracted to CaM’s highly negative (acidic) surface [20,28,44]. Historically, there are four canonical subclasses based on the locations of key hydrophobic residues: 1-10, 1-12, 1-14, and 1-16 [18,45]. The 1-12 (FILVW)xxxxxxxxxxx(FILVW) and 1-16 (FILVW)xxxxxxxxxxxxxx(FILVW) subclasses have no additional variants. The 1-10 and 1-14 do have multiple variants (e.g., 1-5-10: (FILVW)xxx(FAILVW)xxxx(FILVW); 1-5-8-14: (FILVW)xxx(FAILVW)xx(FAILVW)xxxxx(FILVW)). These examples should suffice to explain the basic organization of calcium-dependent binding. Subsequently, other calcium-dependent binding motifs related to canonical binding organization were revealed, including 1-7, 1-17, and a 1-18 in reverse direction, as well as others [20,41,44,46]. While the canonical binding options would seem to be enough to explain how CaM is able to bind to so many targets, other unexpected binding mechanisms have come to light, including calcium-dependent binding to the lipid tail of prenylated proteins (e.g., KRAS4b), to certain free lipids, and to membrane phospholipids [47,48].

Initial research showed CaM wrapped around intrinsically disordered target sequences, as exemplified by binding to myosin light chain kinase (MLCK) and Ca^2+^-CaM-dependent protein kinase II (CaMKII) [20,28]. With wrap-around binding, bending of the flexible linker allowed the N- and C-lobes to entwine an alpha-helical peptide sequence, capturing the hydrophobic anchor residues in the calcium-dependent binding pockets [41,42]. In the case of CaMKII, this displaces an autoinhibitory domain, activating the kinase activity [7]. However, other targets, including the plasma membrane calcium pump or the anthrax edema factor, interact via an extended collapsed binding mode [20,49,50]. Rather than wrapping around or engulfing the target, each lobe binds to spatially separated regions, forcing CaM into a specific active shape. Another observed form of binding involves bipartite or tripartite interactions where each lobe acts independently [51]. Thus, one lobe can bind a target while the other lobe binds to a different domain on that target or on a different protein [52,53]. These binding attributes reveal CaM’s unparalleled flexibility, which is further emphasized by its ability to extend significantly to bind to distant key hydrophobic amino acids in a binding motif.

Ca^2+^-CaM’s longest structural dimension spans between 6.5 and 7.0 nm [28,54]. In its native calcium-bound state, its overall length measures approximately 6.5 nm, but when acquiring an “open clamp” conformation, as observed with HIV-1 matrix (HIV-1 MA) protein target binding, it can extend up to 7.0 nm [55]. It has been calculated that between 18 and 20 amino acids span a linear distance of 7 nm [56]. Of course, the actual distance between two amino acids depends not only on the protein sequence’s shape (i.e., alpha-helix, unfolded polypeptide, beta-sheet) but also on the specific amino acids involved. Furthermore, CaM wraps around most canonical sequences, meaning amino acids that are spaced farther apart than 20 aa could also be subject to binding, such as the C28 region of plasma membrane calcium ATPase PMCA4b [20].

While the calcium-binding EF-hands of CaM are highly conserved (i.e., **D**x**DG**x**G**x[I/V]xxx**E**), they still show variations in calcium binding [43]. The C-lobe has a higher affinity for calcium binding, but the N-lobe binds faster. While each EF hand binds with micromolar affinity and cooperativity occurs between them, C-lobe EF hand-4 (EF-4) binds calcium first, followed by EF-3, then calcium binding to N-lobe EF-2, and EF-1 follows [57,58]. Coupled with this is cooperativity of calcium binding and an increased affinity for calcium that can result upon calcium binding to CaMBPs [59,60].

As might be evident from the differential calcium-binding of its lobes, calcium-dependent CaM binding to targets is not an all-or-nothing response but rather can be thought of as a rheostat function whereby CaM binding changes in response to calcium concentrations [61,62]. As shown for Ca^2+^-activated potassium channels (SK), different targets require specific levels of Ca^2+^-CaM saturation, allowing graded responses in CaM-target binding [63]. Related to this is the involvement of a feedback loop when high levels of Ca^2+^-CaM stimulate clearance via pumps coupled with the shutdown of release channels to reset basal calcium levels [4]. Thus, while the levels of calcium define CaM’s functionality, activated CaM can take control of calcium fluxes to control that target-binding ability as covered in more detail in “Section 4”.

## 4. Calmodulin and Calcium Regulation

Intracellular calcium levels are controlled in part by the influx and efflux of calcium ions via channels, pumps, and receptors in the plasma membrane. Of interest here are those regulated by CaM. Major examples are plasma-membrane Ca^2+^-ATPase (PMCA; pump extrudes calcium), voltage-gated calcium channels (VGCC; channel for calcium influx), vanilloid channel subfamily member 5 (TRPV5; calcium uptake), and N-methyl-D-aspartate receptor (NMDAR; channel for calcium influx) [53,64,65,66,67]. CaM works in different ways to control calcium ion movements in those channels. It should be noted that there are some alternative calcium-dependent feedback and inactivation mechanisms in other channel families that are not mediated by CaM but utilize other calcium-binding proteins or direct binding to the channel itself [68].

To maintain normal cytosolic calcium levels, PMCA uses ATP to pump calcium out of the cell [65,69]. The pump remains inactive in resting cells due to inhibition of the active site caused by the presence of a C-term autoinhibitory sequence within which is located a CaM-binding domain [69,70]. Increasing levels of calcium convert local apoCaM to Ca^2+^-CaM that binds to the autoinhibitory domain, pulling it out of the active site to relieve inhibition and stimulate calcium extrusion [64].

Calcium-dependent inactivation (CDI) is a negative feedback event whereby the influx of calcium ions ends up lowering the activity of the channel through which they enter [66]. Although it varies in channel-specific operational details, CDI functions in cardiac (Cav1.2) and neuronal (Cav1.3) L-type calcium channels, P/Q-type and N-type calcium channels (Cav2.1, Cav 2.2), and NMDAR receptors (via GluN2A and GluN2B subunits) [66,68,71]. The published literature widely accepts the importance of CaM in CDI [53,68,71]. While specifics vary with each channel type and variants based on organ or tissue-specific needs, the pre-association of apoCaM via IQ motif binding sets the stage for a rapid response in each case [53,72]. Acting as the sensor for CDI, apoCaM responds to increasing calcium ion levels, causing its calcium-free lobe to become charged and ready for binding to Ca-dependent binding motifs, causing closure of the ion channel. When normal calcium levels are restored, the events are reversed, and the channel again opens. In keeping with this, it should be acknowledged that calcium cannot directly control CDI; it needs an effector to sense and respond to levels of the divalent cation. Here, the control of CDI by CaM is examined in a non-traditional light wherein CaM is the major regulator of calcium levels, and this regulation is related to its essential to the maintenance of its homeostatic and specialized cellular functions.

## 5. CDI: Another Point of View

**Figure 2:**
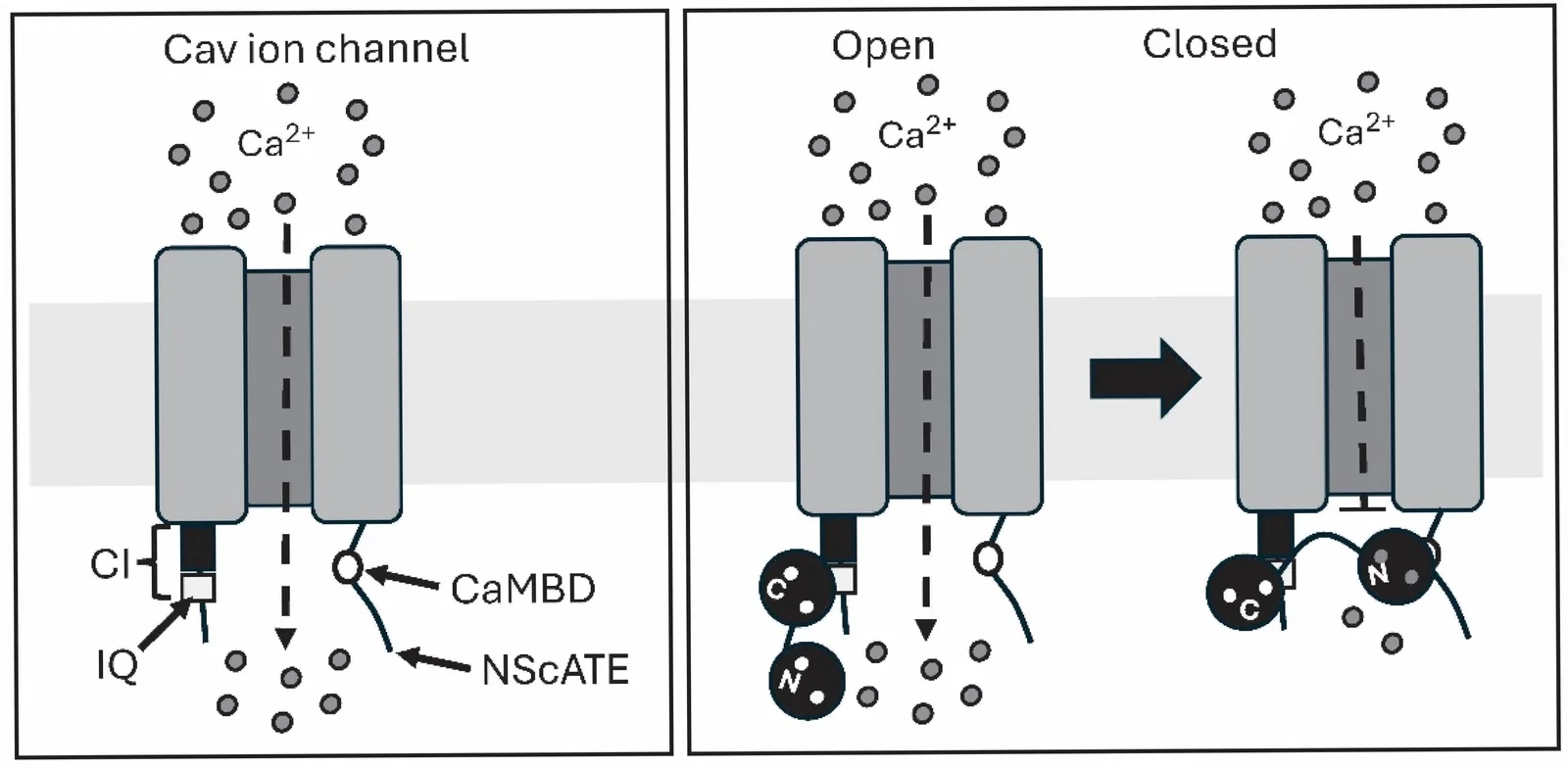
Basic events in Cav1.3 channel regulation by calmodulin. **Left panel**. Illustration of the Cav channel showing the N-term location of a calcium-inhibitory (CI) domain plus an IQ motif for calcium-independent CaM binding. In the C-term, a calcium-dependent CaM-binding domain (CaMBD) and N-terminal spatial Ca^2+^-transforming element (NSCaTE) are present. **Right panel**. In the resting channel-open state, calmodulin is resident on the channel, bound to the IQ motif. As cytosolic calcium levels increase, the N-lobe becomes charged with calcium ions, causing a conformational change in CaM, allowing it to stretch across and bind to the CaMBD. This leads to closure of the channel. This is modified from details in Figures 3 and 4 in [53].

The regulation of voltage-gated CaV1.3 channels serves as a stand-out prototype for the primary events of CDI ion channel regulation (Figure 2) [53]. The carboxy-terminus of the Cav1.3 channels contains a calcium-inhibitory (CI) domain plus an IQ motif. An N-terminal spatial Ca^2+^-transforming element (NSCaTE) is located at the other end. It is an effector site to which Ca^2+^-CaM binds. At low calcium levels, the resting channel is open with resident CaM attached to the IQ motif. When cytosolic calcium levels rise, the N-lobe EF-hands bind calcium, driving a change in shape allowing the N-lobe to reach across and bind to the experimentally validated NSCaTE CaMBD, shutting down the channel.

While this event and its variants are referred to as calcium-dependent inactivation, without CaM, channel closure could not be precisely regulated. Thus, it is a Ca^2+^-CaM-dependent inactivation. Furthermore, there is an additional event involved in CDI: CaM is not just regulating ion flux; it is determining its own functional status. At normal calcium levels, apoCaM dominates the regulatory pattern of CaM targets dominated by IQ motif-containing proteins. At increased calcium levels, Ca^2+^-CaM now shifts to regulate a different group of proteins. The significance of this becomes evident when ion channel regulation is viewed in the light of CaM’s functions in mediating specific events as opposed to maintaining homeostatic calcium levels. In those cases, CaM, not calcium, becomes the central factor regulating calcium levels to efficiently oversee specific functions.

The significance of this interpretation is exemplified by long-term potentiation, a model for learning and memory that is driven by CaM [73,74]. NMDAR is a critical calcium channel that is regulated by CDI and in turn uses the shift in cytosolic calcium levels to control the two key enzymes that act as opposing switches for LTP: CaMKII and calcineurin [74,75]. At resting cytosolic calcium levels (~100 nM), CaM is functionally calcium-free, existing in its condensed form to bind targets typically via IQ motifs. As levels rise (~500 nM to 1 μM), CaM is partially saturated. At this stage, high-affinity targets, including calcineurin, are activated. When saturated levels (>1 μM) of calcium are attained, both lobes are fully charged with the divalent cation. Enzymes, including CaMKII, that require fully loaded CaM are activated at this level. This differential activation of the key CaM-dependent regulators, CaMKII and calcineurin, that depends on calcium ion concentration, is essential to their roles in LTP [43].

The bottom line is that LTP requires enough extended calcium influx coupled with not only the activation of NMDARs by CaM but also its regulation of two primary enzymes involved in models of learning and memory: CaMKII and calcineurin [76]. The complex neuronal interplay involving these components drives LTP and long-term depression (LTD) along with synaptic plasticity. Thus, events of learning and memory require specific events mediated by CaM, events that CaM controls not only through its regulation of NMDARs to control the amount and duration of calcium influx but also by regulating other CaM-regulated effectors (i.e., CaMKII and calcineurin).

To summarize, CaM controls its own ability to bind to and regulate targets by controlling cytosolic calcium levels that drive the shift from its calcium-bound versus calcium-free status. By controlling a diversity of channels and receptors that oversee calcium movements into and out of the cell, CaM can alter local levels of the ion. When calcium levels are low, CaM can direct channels to open, allowing more calcium to flow in. When they are too high, excess calcium can be pumped outside or stored internally. In this way, CaM regulates its own functional status and the CaMBPs to which it can bind and regulate.

## 6. Self-Regulating the *CALM* Genes

Over 20 years ago, it was realized that gene transcription and post-translational covalent modifications are other ways CaM’s activity can be regulated [77]. CaM is encoded in three backup genes: *CALM1*, *CALM2*, and *CALM3* located on human chromosomes 14, 2, and 19, respectively [78,79,80]. Although each of them has a significantly different nucleotide sequence, they all encode identical CaM copies. The *CALM* genetic redundancy serves as a protective backup survival system since the three different sequences would be subjected to different mutational influences.

Despite encoding an identical protein, the different genes are regulated and expressed differently in different cells [80,81]. For example, during sperm formation, each of the three *CALM* genes is expressed at different times. Because each gene is unique, the transcribed RNA also differs. Since the resultant protein is identical, it was necessary to follow the patterns of RNA to assess which genes were active. RNAs for *CALM1* and *CALM3* appear early during mitosis and remain constant throughout meiosis. While *CALM1* RNA continues to be produced only until spermatozoan differentiation begins, *CALM3* gene expression continues until spermatogenesis is completed. *CALM2* RNA expression is limited to early meiosis, where it peaks in amount for a short time before becoming undetectable. While the significance of these changes is unexplained, understanding them could yield additional insight into the significance of having multiple copies of the *CALM* genes.

The three CaM genes are regulated by transcription factors called CAMTAs (CaM-binding transcription activators) [33,82]. As their name implies, CAMTAs bind to and are regulated via CaM binding. CAMTAs are ancient and highly conserved proteins, reflecting their importance in ensuring CaM’s availability. There are two human CAMTAs, *CAMTA1* and *CAMTA2*, which differ in when and where they are activated [33,82]. For example, human CAMTA1 is predominantly found in brain neurons, while CAMTA2 localizes to the heart, muscle, and brain [83]. Minor *CAMTA* mutations result in very small reductions in CaM levels but without altering CaM’s structure. Those small decreases still have deleterious effects. Research in mouse models has shown that mutations in *CAMTA1* cause neurological issues including intellectual problems, decreased memory performance, and neurodegeneration, while overactivation of *CAMTA2* causes heart swelling [84].

Since the levels of CaM are limiting compared to the combined concentration of its targets, small quantitative changes in CaM are predicted to impact CaM-mediated events [85,86]. As a result, minor decreases in CaM levels can have a significant effect on CaM-mediated functions. In support of its ability to self-regulate, when CaM levels drop significantly, they activate the CAMTAs, inducing *CALM* gene expression to re-establish normal levels [82,87]. CAMTAs also shut down *CALM* gene expression when CaM levels get too high. CaM does this by binding to the CAMTAs, inactivating them and preventing gene transcription.

CaM binds to CAMTAs via calcium-independent conserved IQ motifs and calcium-dependent CaMBDs [83]. Since a search of the literature by the author failed to reveal details about the binding, the author used the Calmodulin Target Database to scan the protein sequences of human *CAMTA1* (Q9Y6Y1, 1673aa) and *CAMTA2* (O94983, 1202aa) for IQ motifs and calcium-dependent canonical binding motifs [27]. This revealed that both proteins possess two putative tandem calcium-dependent CaMBDs in their C-term with multiple IQ motifs resident therein. CAMTA1’s first CaMBD (1554VIQRCYRKYKQYALYKKMTQAAILIQSKF1582) has five motifs (1-14, 1-12, 1-10, 1-12, 1-16), while the second CaMBD (1614RQARRTAVIVQQKLRSSLLT1633) contains three motifs (1-12, 1-10, 1-5-10). *CAMTA2* appears to have an extremely long single CaM-binding domain within which are two tandem CaMBDs (1073VAAAVIQRCYRKYKQLTWIALKFALYKKMTQAAILIQS1110) containing 16 separate binding motifs (6 × 1-16, 4 × 1-12, 5 × 1-14, 1-5-10, and 1-10). Although the Calmodulin Target Database, which uses Profile Hidden Markov Model algorithms, is recognized as the gold standard for detecting canonical CaMBDs, their experimental validation as true CaM-binding sites and motifs remains to be done [27,88].

## 7. Calmodulin Controls Its Post-Translational Modifications

Post-translational modifications (PTMs) are changes that occur after the protein has been synthesized that affect protein activity, conformation, interactions with other molecules, localization, stability, and turnover [89]. They can be permanent changes in the protein (e.g., myristoylation) or transient (e.g., phosphorylation). CaM PTMs include acetylation, carboxymethylation, nitration, oxidation, phosphorylation, trimethylation, and proteolytic cleavage. Of interest here are phosphorylation, the most common CaM PTM, and methylation, a major attribute of mature CaM.

Phosphorylating specific amino acids in CaM alters its target regulation. Depending on where it occurs, CaM phosphorylation affects its regulation of ion channels [90,91,92]. Since phosphorylation is a transient, controllable, and repeatable event, this adds another level of regulation to CaM’s regulatory abilities. Furthermore, CaM can control its own level of phosphorylation and thus its ability to control specific cellular events. The phosphorylation of CaM by casein kinase II is an example of this self-regulation. CaM directly binds to and is involved in the inhibitory action of the β-subunit of the Ser/Thr protein kinase casein kinase II [36]. This is a bidirectional relationship since casein kinase II phosphorylates CaM at three sites (Thr79, Ser81, Ser101), each of which impacts CaM’s ability to regulate specific CaMBPs [90]. Thr79 phosphorylation reduces the regulatory response to calcium levels by small conductance Ca^2+^-activated K^+^ channels [93]. CaM’s ability to activate endothelial NOS is reduced by Ser101 phosphorylation [94]. Phosphorylation of Ser81, which occurs in the linker, and Ser101, located in the third EF-hand binding pocket, alters CaM binding, affecting the target profile of the regulator [95].

While CaM’s regulatory role in epidermal growth factor receptor (EGFR) is complex and leads to the induction of downstream signaling cascades, its initial binding to a cytosolic juxtamembrane domain serves in the positive regulation of the receptor [96]. EGF binding activates intrinsic protein-tyrosine kinase activity that drives dimerization and autophosphorylation of the receptor [97]. Under specific conditions that include low calcium levels, the receptor tyrosine kinase activity of EGFR in turn phosphorylates CaM on Tyr99 (pTyr99), an amino acid that is located in the third calcium-binding domain in the C-lobe [98,99]. pTyr99 Ca^2+^-CaM is more effective at activating the EGFR [99]. Thus, by regulating its own PTM phosphorylation, CaM can modify its own activity to ensure its event-specific effectiveness when conditions warrant.

What is especially intriguing is the trimethylation of CaM on Lys115, which is also a self-regulated event. Trimethylation of Lys115 alters its conformational dynamics, thermal stability, and binding to specific targets without broadly disrupting calcium-dependent signaling [100,101]. Trimethylation is catalyzed by CaM-lysine N-methyltransferase (CAMKMT), often referred to simply as CaM methyltransferase [37]. No other substrate has been identified for CAMKMT. The regulation of CAMKMT by CaM involves CaM acting as both a specific substrate and a modulator of the enzyme’s activity [102]. While previous research has not identified the CaMBD, a Calmodulin Target Database scan by the author revealed one putative N-term CaMBD (44APLGAARWKLLRQVLKQKHL53) with two canonical calcium-dependent binding motifs (1-12, 1-5-10). This information could be useful in developing peptide antagonists to learn more about other functions of CAMKMT and its regulation by CaM. Fitting with the localized functions of CaM, the methyltransferase localizes to the cytoplasm and nucleus in the brain, muscle, liver, kidneys, and reproductive systems. Carboxyl methylation, converting enzyme proteolytic cleavage, and amino-terminal acetylation are reversible PTMs of CaM that have temporary effects on its functionality but have not been shown to involve CaM autoregulation [103].

## 8. Discussion

**Figure 3:**
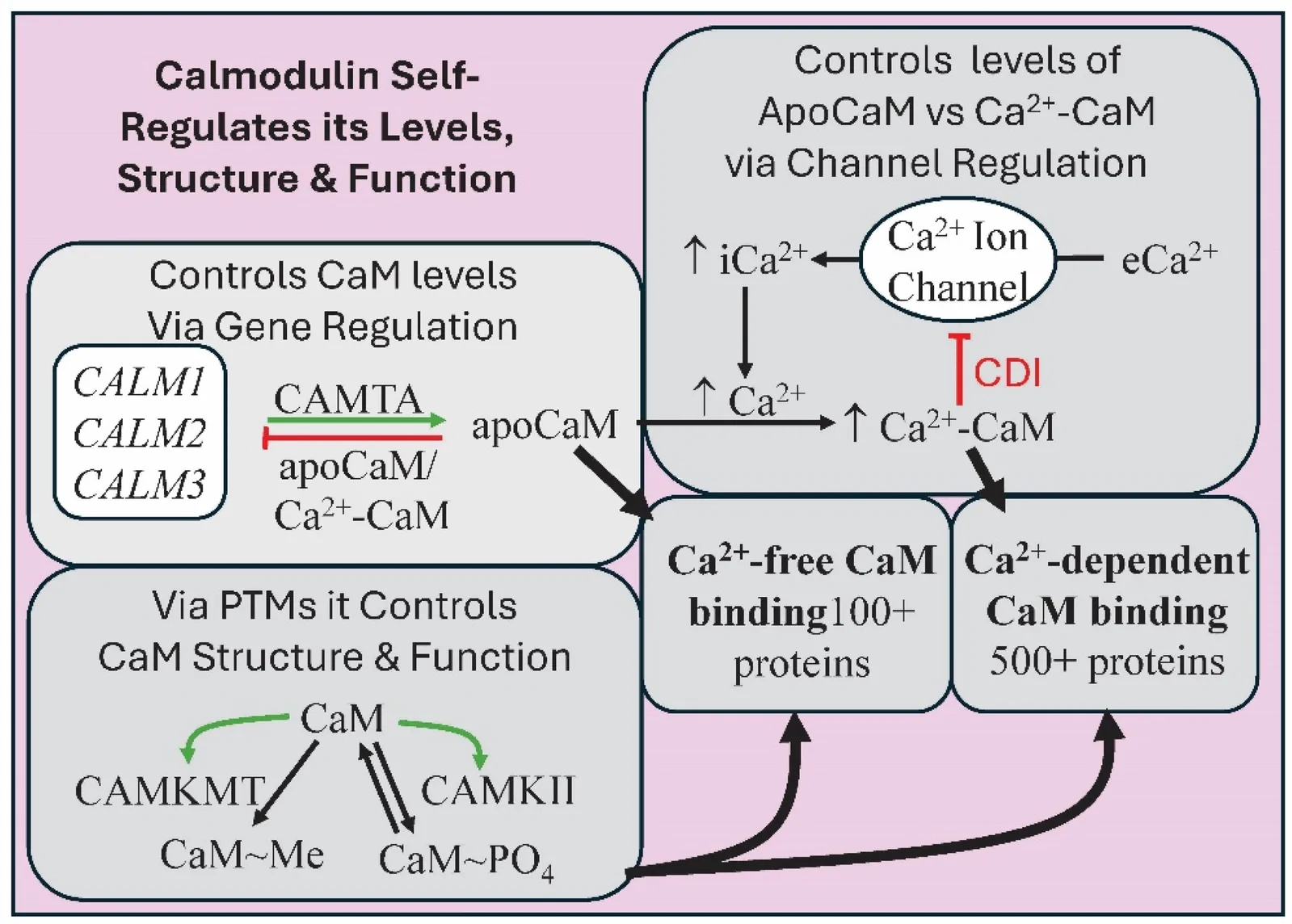
Calmodulin controls its own activation, amount, and functionality via the regulation of calcium ion channels, CAMTA control of calmodulin gene (*CALM*) expression, and post-translational modifications (PTMs). Acting independently or together, these affect its calcium-free and calcium-bound target-binding ability, its levels, and its functionality as detailed in the main text. Acronyms: CaMKII, calcium–calmodulin-dependent protein kinase II; CAMKMT, CaM-lysine N-methyltransferase; CAMTA, CaM-binding transcription activators; CDI, calcium-dependent inactivation.

As our understanding of cell structure and function continues to evolve, it is important to re-examine how different cellular events interact as part of the whole cell ecosystem. Here, CaM has been re-evaluated as an essential biological regulator that self-regulates its own activity, structure, and function. This is not to imply that CaM is a sentient molecule that goes by different rules than other proteins, but to reflect on its importance in cell function and how its critical roles are maintained and assured by the protein’s ability to regulate itself. As summarized in Figure 3, this conclusion was reached by reinterpreting the interactions involving CaM as a regulator of specific examples of CDI, of CAMTA regulation of *CALM* gene expression, and CaM PTMs of phosphorylation and methylation. These events of self-regulation fit with CaM as an essential protein involved in controlling primary life processes.

Found in animals, plants, fungi, and microbes, evidence that CaM is an essential protein comes from many sources, including its cellular and physiological necessity and evolutionary and genetic conservation [5,104,105,106]. An early mutational study on CaM function in the yeast *Saccharomyces cerevisiae* revealed that cells could grow and function with site-directed mutations to the calcium-binding lobes, but CaM null mutations were lethal [107]. Since then, mutations in the C-lobe and adjacent regions of CaM have been revealed to cause heart arrhythmias, but no viable CaM null mutations have been revealed [108]. Coupled with this, various lines of evidence reveal that eukaryotes have multiple means designed to protect against mutational loss.

The fundamental structure and functional attributes of CaM have not changed over millions of years such that microbial and human CaM are virtually identical. Coupled with this is its encoding in three distinct genes (*CALM1*, *CALM2* and *CALM3*), each with different nucleotide sequences that generate an identical end-product [80,81]. This genetic redundancy protects against mutational loss. Furthermore, the *CALM* genes are among the most mutation-intolerant sequences in the human genome [109]. The genes are regulated by *CAMTA1* and *CAMTA2*, ancient and highly conserved proteins, emphasizing their significance in ensuring CaM’s availability [33,82]. Furthermore, their regulation reveals a critical aspect of CaM’s ability to self-regulate its own expression levels. Both CAMTAs are calcium-dependent CaMBPs [83]. Evidence that CaM levels are self-regulated is supported by evidence that decreases in CaM protein activate the CAMTAs, inducing *CALM* gene expression to re-establish CaM levels, but excess CaM levels activate CAMTAs, shutting down *CALM* gene expression [82,87].

In addition to self-controlling appropriate expression levels of protein, CaM also controls its own regulatory abilities by affecting its apoCaM-Ca-CaM transition through the control of cytosolic calcium concentrations. A number of calcium channels and receptors are controlled by CDI, including cardiac (Cav1.2) and neuronal (Cav1.3) L-type calcium channels, P/Q-type and N-type calcium channels (Cav2.1, Cav 2.2), and NMDAR receptors (via GluN2A and GluN2B subunits) [66,68,71]. While calcium has historically been considered the controller of CDI, the information presented here clearly supports the concept that CDI is fundamentally under the control of CaM. Furthermore, important to the theme of this article is that via CDI, CaM is able to determine its own functional state, controlling whether it operates as apoCaM with its specific calcium-free IQ-dominated target population or charged as Ca-CaM that binds to a larger, alternative calcium-dependent set of CaMBPs. The implications of this self-regulation were exemplified by CaM’s role in regulating the contradictory actions of CaMKII and calcineurin in LTP [73,74].

CaM also fine-tunes its regulatory abilities via its control over post-translational modifications, including an initial, sustained methylation and subsequent transitory shifts in phosphorylation [37,91,92]. Trimethylation on Lys115 is catalyzed by CAMKMT, a CaM-regulated methyltransferase [37]. Thus, the early PTM that stabilizes CaM structure and impacts its functions is under its own control. CaM’s transient phosphorylation status has a significant impact on its routine and specialized signaling functions, with the site of phosphorylation impacting the events it regulates. A number of CaM-regulated kinases (e.g., casein kinase II; EGFR) control which of CaM’s amino acids are phosphorylated, thus evidencing how CaM has control of its own regulatory actions [36,90,91].

## 9. Conclusions

The novel revelations about CaM’s ability to regulate aspects of its activity, structure, and function offer new ways to look at its significance and potential as a therapeutic target in a diversity of diseases. While this review has implications for our fundamental understanding of CaM function and regulation, it also suggests that future avenues of research need to consider many of the issues covered. Because mutations in each of the three *CALM* genes can cause heart arrhythmias, understanding their individual expression levels could help explain phenotypic severity of patient calmodulinopathies and potential therapeutic routes to dealing with them [79,80]. In mouse cardiomyocytes, there is a differential expression of the three CALM genes with the different cytoplasmic locations potentially related to functional CaM availability in normal and diseased myocyte functions [80]. In humans, there are tissue-specific variations in the expression of each gene as well as variations in the pathogenicity of missense mutations in *CALM1* and *CALM2* versus *CALM3* [79]. Thus, understanding more about how CaM controls itself and its gene-specific expression levels may offer novel routes to dealing with calmodulinopathies, for example.

CaM is intimately involved in the earliest events of Alzheimer’s and other neurodegenerative diseases, and potential therapeutic routes based on peptide targeting of CaM-binding motifs have been proposed [110]. That being said, specific information on CaM remains undone [6,110]. An early immunological study suggested that CaM levels are reduced in AD brains, but that work used less-specific antibodies than are available today. More importantly, PTMs linked to CaM function have never been quantified or localized in neurodegenerative diseases (NDDs). Their impact on CaM’s critical involvement in both amyloidogenesis and Tau phosphorylation could provide new therapeutic routes [111]. Expression levels of the three CALM genes and the two CAMTAS that regulate them need to be studied, since they could provide treatments designed to re-establish CaM levels should they be proven to decline in critical brain regions during AD and other NDDs. Considering the importance of CaM in basic biological events, as well as in a wide diversity of diseases, coupled with new insights into its self-regulation, it is time for more detailed research on this master protein to be conducted.

## Data Availability

No new data were created or analyzed in this study. Data sharing is not applicable to this article.
